# Liposomal Tubacin: Strategies for the Formulation of a Highly Hydrophobic Anticancer Drug

**DOI:** 10.3390/pharmaceutics17040491

**Published:** 2025-04-08

**Authors:** Cindy Schelker, Léa Revaclier, Gerrit Borchard, Patrycja Nowak-Sliwinska

**Affiliations:** 1 School of Pharmaceutical Sciences, Faculty of Science, University of Geneva, 1211 Geneva, Switzerland; cindy.schelker@unige.ch (C.S.);; 2 Institute of Pharmaceutical Sciences of Western Switzerland, University of Geneva, 1211 Geneva, Switzerland; 3 Translational Research Center in Oncohaematology, 1211 Geneva, Switzerland

**Keywords:** design of experiment, drug delivery, histone deacetylase inhibitors, liposomes, Plackett–Burmann, tocopheryl polyethylene glycol succinate

## Abstract

**Background:** Clear-cell renal cell carcinoma (ccRCC) is the most prevalent form of kidney cancer, accounting for over 75% of cases worldwide. Histone deacetylase inhibitors (HDACIs) have emerged as promising agents for ccRCC treatment, particularly in combination with immunotherapy or targeted therapies. Tubacin, a potent HDAC6 inhibitor, has demonstrated potent anticancer activity but faces therapeutic limitations due to its hydrophobic nature and poor solubility, which hinder its effective drug delivery. This study explores liposomal encapsulation as a strategy to improve tubacin delivery; **Methods:** Liposomes were prepared using the ethanol injection method followed by size-exclusion chromatography. Using the Plackett–Burman Design, we identified a promising liposomal formulation and evaluated its biological activity in vitro; **Results:** However, initial formulations reduced the mitochondrial activity to 30% in healthy renal cell lines. To mitigate this, we optimized the formulation by reducing tocopheryl polyethylene glycol succinate (TPGS) content and incorporating Kolliphor^®^ as an additional surfactant. This optimized formulation significantly reduced toxicity in noncancerous cells, with up to 80% of mitochondrial activity conserved while retaining key properties for therapeutic application; **Conclusions:** Our findings demonstrate that liposomal encapsulation enhances the safety and delivery of hydrophobic drugs like tubacin. This approach offers a promising strategy for improving the efficacy of HDACIs in ccRCC treatment, potentially overcoming drug delivery challenges associated with hydrophobic molecules.

## 1. Introduction

Clear-cell renal cell carcinoma (ccRCC) is the predominant kidney cancer type, with 75% of cases worldwide [1]. Targeted drug therapy employs tyrosine kinase inhibitors (TKIs) such as pazopanib and axitinib, both targeting angiogenesis-related mechanisms [2]. Various drug combinations of established and newly approved targeted drugs are being explored [3,4]. However, most drug combinations lack specificity, resulting in pronounced toxicity and reduced safety [5]. Well-designed anticancer drug combination therapies can target multiple pathways, therefore causing diminished acquired drug resistance development. By taking advantage of drug–drug interaction between two or several drugs, a lower individual drug dose is needed, reducing side effects and increasing safety. In previous studies, we used the Therapeutically Guided Multidrug Optimization (TGMO) method [6,7,8] on ccRCC cells to identify a synergistic four-drug combination called C2. The C2 was highly selective in ccRCC cells at high efficacy [9]. The C2 combination consisted of two TKIs, i.e., erlotinib.HCl and dasatinib, and two histone deacetylase inhibitors (HDACI), i.e., tacedinaline and tubacin. The most potent drug–drug synergism was observed between tubacin and erlotinib.HCl.

Overexpression of HDACs in ccRCC is documented to lead to cell invasion, with HDAC6 decreasing the expression of acetylated α-tubulin, resulting in an enhancement of cell motility [10]. In a phase I/II clinical trial, entinostat was combined with interleukin-2, and objective response rates (ORR) were obtained at 37% with progression-free survival (PFS) of 13.8 months versus 25% of ORR and 4.2 months of PFS with IL-2 treatment as monotherapy [11]. This study was the first to document the improvement in a benefit of immunotherapy for ccRCC. In a phase I/II clinical trial, vorinostat, an inhibitor that targets class I, II, and IV HDACs, when combined with bevacizumab, a recombinant humanized monoclonal antibody that binds and neutralizes circulating vascular endothelial growth factor, led to a clinical benefit, with 48% of patients having prolonged PFS and improved overall survival compared to a bevacizumab-only study arm [12]. These outcomes are the basis of a solid rationale for the incorporation of HDACIs in drug combinations against ccRCC.

In the current study, we focused on the encapsulation strategies of tubacin, an HDAC6 inhibitor. Although tubacin remains an investigational drug, its anticancer properties have been documented in several papers [13,14,15,16]. Tubacin was reported to be effective in bladder cancer where a gene encoding fibroblast growth factor receptor 3 (FGFR3) is mutated and contributes to cancer progression. HDAC6 inhibition by tubacin leads to degradation of mutant FGFR3 and significantly reduces tumor growth [13]. Tubacin was also reported to be active against acute lymphoblastic leukemia (ALL) through increasing acetylation of α-tubulin, leading to microtubule stabilization [14], which, in turn, led to the ubiquitinated accumulation of misfolded proteins. These mechanisms led to apoptosis of ALL cells both in vitro and in vivo [15]. In another study on triple-negative breast cancer where high expression of HDAC6 is linked to tamoxifen resistance, HDAC6 deacetylates the heat shock protein 90 (HSP90), essential for the correct folding of oncogenic proteins. Tubacin treatment causes a hyperacetylation of HSP90 and its loss of activity, resulting in growth suppression of tamoxifen-resistant cells in vivo [16]. However, tubacin is highly hydrophobic with a log P of 7. This poor solubility and, thus, the necessary exposure to a high concentration to obtain in vivo responses limit its potential clinical use [13].

One common strategy to overcome the poor aqueous solubility of an active pharmaceutical ingredient (API) is its encapsulation within a nanocarrier. Delivery of APIs in nanocarrier formulations decreases off-target effects and increases the therapeutic index of the API [17,18]. One example of this application is the encapsulation of doxorubicin, an API known for its dose-dependent cardiotoxicity, which was overcome by incorporating it into liposomes, effectively reducing the cardiotoxic effects of the free drug [19].

Anticancer lipid-based nanoparticles, especially liposomes, represent the highest number of FDA-approved anticancer drugs [20]. They offer features such as the possibility of active targeting or triggering drug release by specific stimuli [21]. Among other important features, liposomes also allow sustained release of the API, allowing for longer exposure of the body to the API and reducing rounds of administration. A milestone in liposomal development was the introduction of Doxil^®^/Caelyx^®^ for the treatment of ovarian cancer. The FDA approved the first liposomal formulation of doxorubicin in 1995, reducing its cardiotoxicity and, thus, increasing safety [22].

One of the drawbacks frequently addressed in liposomal formulation research is their laborious development. The path of liposomal development is not linear and there is a lack of defined protocols that can be applied to every case. A common approach used by formulation scientists is the reliance on other known strategies as a base. One can think of the use of long and saturated acyl chains for their main phospholipids to raise bilayer transition temperature and thus increase particle stability [23]. Another option is the addition of 30 %mol of cholesterol to decrease bilayer fluidity and drug leakage [24,25]. These strategies are still rather scarcely applied and may not be applicable to every formulation. As a result, the development of a formulation at the screening-stage level by varying factors individually can be tedious [26]. Among variables to select and test, excipients and manufacturing parameters are the two main categories.

Conventional formulation screening methods involve optimizing one variable at a time while keeping all other variables constant. This rather lengthy and costly process gives less feedback on the correlation between the inputs and outputs. Therefore, the use of an experimental design able to screen multiple variables simultaneously with the lowest number of experimental iterations should be a tool implemented in every development step. The aim is to balance between a minimal number of iterations and obtain an acceptable accuracy of responses. To mitigate this, we employed a design of experiment (DoE). The quality by design (QbD) reduces experimental efforts while increasing the usefulness of the data gathered [27]. This approach can help to identify significant factors and their impact, both positive and negative, on a response, in our case, an encapsulation efficiency or example size.

In this work, we used a DoE, more specifically the Plackett–Burmann Design (PBD) to determine the optimal composition and synthesis parameters for liposomes designed to encapsulate tubacin. The PBD is a two-level experimental design, meaning each variable is tested at two different values. This approach enables the identification of linear relationships between variables and responses while minimizing the number of experimental runs required [28].

## 2. Materials and Methods

### 2.1. Materials

Tubacin (purity ≥ 98% HPLC), dipalmitoylphosphatidylcholine (DPPC), distearoylphosphatidylcholine (DSPC), cholesterol, tocopheryl polyethylene glycol succinate (TPGS), cholesterol, Kolliphor^®^, Triton X-100^®^, anhydrous DMSO, and phosphate salt (H_2_KO_4_P) were purchased from Sigma Aldrich Chemie GmbH (Schnelldorf, Germany). Methanol, acetonitrile, and ethanol were obtained from Fischer Chemical (Reinach, Switzerland). A Sephadex G-25 Hitrap desalting column was purchased from Cytiva (Rosersberg, Sweden) and an electronic pipette was from Eppendorf (Hamburg, Germany).

### 2.2. Physicochemical Properties of Liposomes

#### 2.2.1. Plackett–Burmann Design (PBD)

The PBD-based screening table was constructed using the software Design Expert v13 (Minneapolis, MN, USA). Both formulation- and manufacturing-process-related variables selected for the screen are presented in Supplementary Table S3. The levels of each variable were selected based on the literature and preliminary studies. Concerning temperature, the levels selected were chosen according to the work of Muthu et al. [29], where the authors used an identical lipid composition for a liposomal formulation. Stirring speed and loading time were shown as factors affecting particle size [30]. Tubacin and lipid concentrations were found to have an impact on encapsulation, size, and polydispersity index (PDI) in previous experiments. Finally, DMSO was used as a co-solvent for solubilizing in tubacin in ethanol, making its concentration a relevant parameter to investigate.

#### 2.2.2. Liposome Preparation

Liposomes were prepared using the ethanol injection method. Depending on the Plackett–Burman Design, different concentrations of tubacin (0.02 or 0.05 mg/mL), DPPC (0.6 or 1 mg/mL), cholesterol (0.4 or 1 mg/mL), TPGS (0.2 or 0.6 mg/mL), and DMSO (0.2 or 0.5 mL) were dissolved in 1 mL of heated ethanol. The aqueous phase, formed by 0.9% NaCl at pH 7, was heated at a temperature (either 50 or 60 °C). The suspensions were prepared by injecting 1 mL of ethanol phase in 4 mL of the aqueous phase at a controlled flow rate using an electronic pipette (Eppendorf) under magnetic stirring (600 or 800 rpm). After liposome formation, stirring was continued during a defined time (5 or 10 min) according to the Plackett–Burmann Design. The suspension was then put on ice for 1 h. Free tubacin, ethanol, and DMSO were removed with a Sephadex G-25 column using 0.9% NaCl, pH 7, and filtered with 0.22 µm PVDF filters as the elution buffer. The liposome suspensions were stored at 4 °C.

For the preparation of Formulation I, 50 µL of tubacin (5 mg/mL), DPPC (0.6 mg/mL), cholesterol (1 mg/mL), and TPGS (1 mg/mL) were dissolved in 1 mL of ethanol heated at 60 °C. The ethanolic phase was injected in 4 mL of NaCl 0.9% under magnetic stirring at 600 rpm at 60 °C. After liposome formation, stirring was continued for 5 min. The suspension was then put on ice for 1 h. Free tubacin, ethanol, and DMSO were removed with a Sephadex G-25 column using 0.9% NaCl, pH 7, and filtered with 0.22 µm PVDF filters as the elution buffer. The liposome suspensions were stored at 4 °C.

For Formulation II, the preparation method was similar, with differences in the lipids and their concentrations. A total of 50 µL of tubacin (5 mg/mL), DSPC (0.3 mg/mL), cholesterol (0.1 mg/mL), and TPGS (0.2 mg/mL) were dissolved in a heated mixture of Kolliphor^®^ HS-15 at a concentration of 0.04 mg/mL in 1 mL of ethanol. The rest of the preparation was identical to Formulation I. Finally, liposome suspensions were freeze-dried until further analysis.

#### 2.2.3. UHPLC Instrumentation and Chromatographic Conditions

For Formulation I and II, the chromatographic separation of tubacin was conducted using a Waters Acquity System (Milford, MA, USA) equipped with a binary solvent delivery pump, autosampler, sample manager, and a photodiode array (PDA) detector. Separation was carried out on an acquity UPLC^®^ BEH C18 2.1 × 100 mm, 1.7 µm column. The column was heated to 30 °C. The mobile phase was composed of solvent A (phosphate buffer: H_2_KO_4_P 0.05M, pH 6.5 adjusted with NaOH) and solvent B (methanol/acetonitrile 70:30). A gradient method was used starting from a mixture of 50% of A and 50% of B to 100% of B in 6 min. From 6 min up to 6.1 min, the mobile phase was a mixture of 50% A and 50% B and stayed at this composition for 7 min. The flow rate was set to 0.3 mL/min and UV detection was achieved at 246 nm. The injection volume was 10 µL. Stock solutions of tubacin (5 mg/mL) were prepared in DMSO and stored at −20 °C until further use. Calibration solutions of tubacin were prepared under the same conditions as the samples. An example of a chromatograph of tubacin separation with UPLC-UV and the table with data extracted from the chromatogram can be found, respectively, in Supplementary Figure S1 and Supplementary Table S1. An example of a calibration curve of tubacin in NaCl/DMSO 1:4 *v*/*v* is displayed in Supplementary Figure S2.

#### 2.2.4. Determination of Encapsulation Efficiency (EE%)

Quantification of encapsulated tubacin in liposomes was performed using the UPLC method described above. Briefly, 1 µL of Triton X-100 was added to 200 µL of liposome suspension and vortexed for 10 s. The mixture was heated at 50 °C in an ultrasound bath for 15 min for complete liposome disruption. Samples were diluted with NaCl/DMSO 1:4 and injected for UPLC analysis.

The EE% is obtained with the equation: EE% = $\frac{Tubacin\;encapsulated}{Tubacin\;initial\;input}\times 100$.

#### 2.2.5. Batch-Mode Dynamic Light Scattering (DLS)

Determination of the hydrodynamic diameter and polydispersity index (PDI) was conducted by dynamic light scattering (DLS) using a zeta sizer (NanoZS, Malvern Panalytical, Malvern, UK) in batch mode. Samples were measured at 25 °C in polystyrene disposable cuvettes. The measurement angle was 173°, the refractive index was set at 1.345, and the absorption at 0.010. The laser attenuator was adjusted automatically. Measurements were performed in triplicates. The equilibration time between samples was 120 s. Size and PDI values were measured after dilution at a ratio of 1:20 with NaCl 0.9% filtered through filters of a 0.22 µm pore size. Data were collected using Zetasizer software v7.13.

#### 2.2.6. Asymmetrical Flow Field Flow Fractionation (AF4)

AF4 measurement was performed exclusively for Formulation I using AF4 system (AF2000 system, Postnova Analytics, Landsberg, Germany). The system consisted of an autosampler (PN5300), with a solvent organizer (PN7140), a degasser (PN7520), and smart stream splitter (PN1650), an FOC pump (PN1130), and a TIP pump (PN1130). The separation channel was equipped with a 10 kDa regenerated cellulose membrane with a 350 µm spacer. The system was connected to four online detectors, i.e., a refractive index detector (PN3150), a Multi-Angle Light Scattering (MALS) detector (PN3609), a UV/Vis (Waters 2487) detector measuring at a wavelength of λ = 246 nm, and the DLS system (NanoZS, Malvern Panalytical, Malvern, UK). The mobile phase consisted of the same external liposome buffer (0.9% NaCl filtered through 0.1 µm pore size filters). For flow-mode DLS analysis, measurements were obtained with a quartz flow cell (ZEN0023, Malvern Panalytical, Malvern, UK). The gyration radius analysis was performed by the MALS detector using a sphere fit model. The detector flow rate was 0.5 mL/min, with a focus step delay time of 3 min. The injection flow was 0.20 mL/min, with an injection time of 7 min. The crossflow was 1 mL/min and the focus pump was at 1.30 min. Elution step parameters are described in Supplementary Table S4. Finally, the rinse step had a TIP flow of 0.05 mL/min and a focus flow of 0.05 mL/min.

#### 2.2.7. Freeze-Drying Process

The freeze-drying process was performed on Formulation II. A total of 1 mL of liposome suspension was freeze-dried with sucrose 10%. Briefly, 800 µL of liposomes and 200 µL of sucrose 50% were mixed and freeze-dried following the protocol presented in Supplementary Table S2.

#### 2.2.8. Transmission Electron Microscopy (TEM)

Lamellarity, shapes, and sizes were assessed by TEM. Carbon hexagonal mesh with 200 copper grid mesh was used (Electron Microscopy Science, Hatfield, PA, USA). They were glow-discharged using an EMS GlowQube instrument. Samples were diluted with NaCl 0.9% solution at a ratio of 1:10. A total of 5 µL of the sample was deposited on the grid. Samples were left on the grid for 30 s, after which the surplus was removed with absorbent paper. Uranyl acetate 1% was used for liposome staining. Micrographs were acquired with a G2 Sphera microscope (Thermofisher, Waltham, MA, USA) operating at 120 kV, with a defocus range of −1 to −2 µm. Image analysis was conducted using Fiji software (ImageJ, 1.53f51). Both Formulation I and II were analyzed by TEM.

### 2.3. Biological Characterization of Liposomes

#### 2.3.1. Cell Culture

Human embryonic kidney 293T cells (HEK-293T), human clear-cell RCC cell line (786-O), and renal proximal tubule epithelial cells (RPTEC) were purchased from American Type Culture Collection (ATTC, Teddington, UK). The cells were cultured in RPMI 1640 medium supplemented with 10% (*v*/*v*) of heat-inactivated fetal bovine serum (FBS) and 0.1% of penicillin/streptomycin (Gibco, Thermofisher, Carlsbad, CA, USA).

#### 2.3.2. Mitochondrial Activity (WST-1)

The mitochondrial activity of Formulation I was evaluated on HEK-293T, 786-O, and RPTEC cells. For Formulation II, it was evaluated on HEK-293T and 786-O cells. Cells were seeded at an initial density of 5 × 10^5^ cells per well in 96-well plates and allowed to attach for 24 h. Cells were treated with either 0.9% NaCl in cell culture medium as a control for “blank liposomes” and “liposomal formulation of tubacin” (CTRL) or 0.9% NaCl in cell culture medium with 0.1% DMSO as a control for the “free tubacin” condition. Treatments included blank liposomes, free tubacin, or liposomal tubacin at 5 µM (Formulation I or II) and incubated for 24 h. After incubation, the medium was aspirated and 100 µL of WST-1 reagent (4-[3-(4-iodophenyl)-2-(4-nitrophenyl)-2H-5-tetrazolio]-1,3-benzene disulfonate, Roche, Basel, Switzerland) diluted 1:10 with medium was added to each well. The cells were incubated for 30 min in a cell incubator at 37 °C in a humidified atmosphere with 5% CO_2_. UV absorbance was measured at λ = 450 nm using a microplate reader (BioTek Instruments, Sursee, Switzerland). The CTLR condition was used as a positive reference, corresponding to 100% viability. Equation (1) was used to calculate the mitochondrial activity of every sample:

(1) $$\%Mitochondrial\;activity=\frac{Absorbance\;\left(sample\right)}{Absorbance\;(Untreatred\;sample)}\times 100$$

The cytotoxicity of Formulation I only was also evaluated with an LDH assay. The methodology can be found in the Supplementary Information S1.

#### 2.3.3. Albumin Interaction

Albumin interaction was studied exclusively with Formulation II. Bovine serum albumin (BSA) solutions were prepared in PBS at pH 7.4 without calcium and magnesium. Solutions with concentrations of 5 and 30 mg/mL were prepared. The BSA solutions were incubated with liposomes for 1 h at 37 °C under continuous stirring. UV absorbance was measured across wavelengths from 230 to 700 nm. The fluorescence emission spectrum was recorded following excitation at λ_ex_ = 280 nm. Both measurements were obtained using a microplate reader (BioTek Instruments, Sursee, Switzerland).

#### 2.3.4. Immunofluorescence

Immunofluorescence was studied for Formulation II. 786-O cells were seeded at an initial density of 10^6^ cells/well in 6-well plates. Twenty-four hours after seeding, they were treated with 5 µM and 10 µM of free tubacin solution in RPMI medium, 5 µM equivalent of liposomal tubacin (Formulation II), and blank liposomes. The negative control consisted of a complete medium supplemented with an equivalent volume of 0.9% NaCl, corresponding to the volume used in the liposome treatment. After 2 h of treatment, the cells were fixed using paraformaldehyde 4% (Bio-Rad, Cressier, Switzerland) in calcium- and magnesium-free PBS. Permeabilization and blocking steps were then performed using 2% BSA (Gibco, Thermofisher, Carlsbad, CA, USA) and 0.1% Triton-X100 in PBS. Immunostaining was performed with a primary antibody anti-alpha tubulin (ab179484, Abcam, UK) and a secondary antibody (goat anti-mouse Alexa fluor 488) (Thermofisher, Carlsbad, CA, USA). A total of 20 µL of Vectashield^®^ mounting medium containing DAPI (Reactola, Servion, Switzerland) was added to the samples. Fluorescence readouts were performed with a Biotek Citation 3 (BioTek Instruments, Sursee, Switzerland) with the corresponding software at default settings.

#### 2.3.5. Western Blot

Using Formulation II only, a Western blot assay was performed. 786-O cells were seeded at a density of 10^6^ cells/well in 6-well plates. After 24 h of seeding, they were treated with a solution of 5 µM of free tubacin in RPMI, 5 µM of tubacin loaded in liposomes (Formulation II), and blank liposomes. The negative control was a complete medium supplemented with an amount of 0.9% NaCl corresponding to the volume of 0.9% NaCl used in the liposome treatment. After 2 h of treatment, cells were lysed with radioimmunoprecipitation assay (RIPA) lysis buffer (Thermofisher, Carlsbad, CA, USA). Cell extracts were separated by SDS-polyacrylamide gel electrophoresis (Bio-Rad, Hercules, CA, USA) and transferred to nitrocellulose membrane. Acetylated alpha-tubulin was incubated with the primary antibody (mouse monoclonal, ab179484, Abcam, UK) overnight at 4 °C under gentle agitation. The primary antibody was detected using a secondary antibody (goat anti-mouse, ab195887, Alexa fluor^®^ 488, Abcam, UK) and visualized using Odyssey^®^ imaging system (Li-Cor Biosciences, Lincoln, NE, USA). The signal intensity for each protein was normalized to the corresponding vinculine signal. The original gel is presented in Supplementary Figure S6.

#### 2.3.6. Statistical Analysis

Data analysis was performed using GraphPad Prism software version 10.2.3. Comparisons of the data between groups were performed with the use of a two-way ANOVA with Tukey’s multiple comparisons test. All *p* values < 0.05 were considered statistically significant.

## 3. Results and Discussion

### 3.1. Plackett–Burmann Design

Using the Plackett–Burmann Design (PBD), we evaluated nine formulations to pinpoint the critical variables that could significantly influence the EE%, particle size, or PDI.

Table 1 shows details of all nine formulations included in the DoE. EE% varied between 9% (Formulation 9) and 38% (Formulations 4 and 7). The Pareto chart representing the significance level and type of impact (positive/negative) on the EE% is presented in Supplementary Figure S7. Sizes measured by batch-mode DLS (hydrodynamic diameters) ranged between 130 ± 4 nm (Formulation 8) and 236 ± 24 nm (Formulation 1). The Pareto chart for each parameter’s impact on size is displayed in Supplementary Figure S8. The PDI remained between 0.066 (Formulation 5) and 0.174 (Formulation 1) for all nine formulations. Values obtained varied between 0.066 (Formulation 5) and 0.174 (Formulation 1). The Pareto chart illustrating the significance levels of parameters on PDI is provided in Supplementary Figure S9.

The *p*-value of each variable’s effect on responses is summarized in Table 2. For the EE%, DPPC, TPGS, and DMSO, quantities were all significant. DPPC and DMSO reduced tubacin encapsulation, while TPGS caused an increase in drug encapsulation.

Particle size was significantly influenced by rotation speed, tubacin concentration, and DMSO. Specifically, both rotation speed and tubacin concentration were associated with an increase in particle size, whereas DMSO resulted in a size reduction. In contrast, the model for PDI was statistically insignificant, suggesting that none of the investigated parameters exerted a significant effect on PDI.

### 3.2. Formulation I Development: Selection of Optimal Tubacin-Loaded Liposome Formulation

Formulation 4 satisfied the target product profile with the highest EE% and with particle sizes of 135 ± 8 nm, within the range of 100–150 nm. Therefore, it was selected for further development. As previously discussed, both DPPC input and DMSO were associated with a reduction in encapsulation efficiency. Consequently, the DPPC amount was maintained at the lowest tested level (corresponding to 3 mg input), while DMSO was eliminated from the formulation. Temperature, cholesterol concentration, stirring speed, and time were kept consistent with the conditions used for Formulation 4.

**Figure 1:**
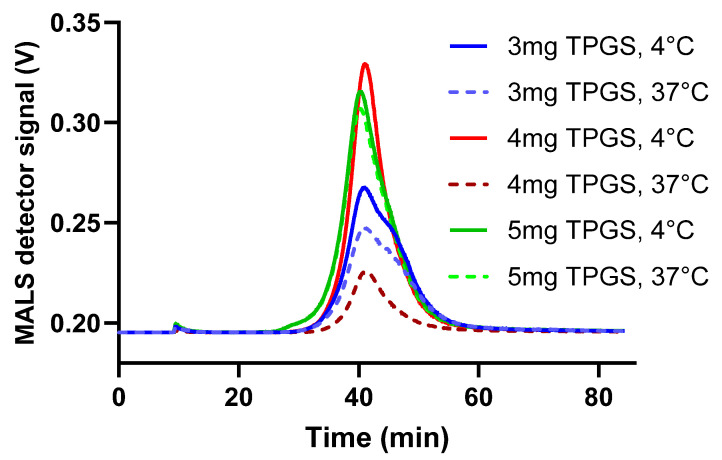
AF4-DLS-MALS fractograms were generated for formulations containing varying concentrations of TPGS. The sample manager was maintained at either 4 °C to simulate the fridge storage conditions or at 37 °C to mimic body temperature. The data are represented as mean ± SD (N = 1).

To evaluate the impact of TPGS on size variability, formulations were prepared with higher TPGS mass inputs of 3 mg, 4 mg, and 5 mg. These formulations were labeled as 3 mg TPGS, 4 mg TPGS, and 5 mg TPGS, respectively, for testing purposes. The size stability of the formulations was assessed at both 4 °C and 37 °C using AF4-DLS-MALS. As shown in Figure 1, the fractograms for 3 mg TPGS at both temperatures and for 4 mg TPGS at 37 °C exhibited two distinct peaks, suggesting the presence of at least two populations with different sizes. The 5 mg fractogram exhibited one peak at both temperatures, indicating enhanced stability across temperatures. Consequently, the formulation 5 mg TPGS was designated as Formulation I. A detailed description of the parameters characterizing Formulation I is provided in Table 3.

### 3.3. Characterization of Formulation I and II

In the next step, Formulation I and II were characterized for their Z-average, PDI, EE%, storage stability, and biological toxicity.

**Figure 2:**
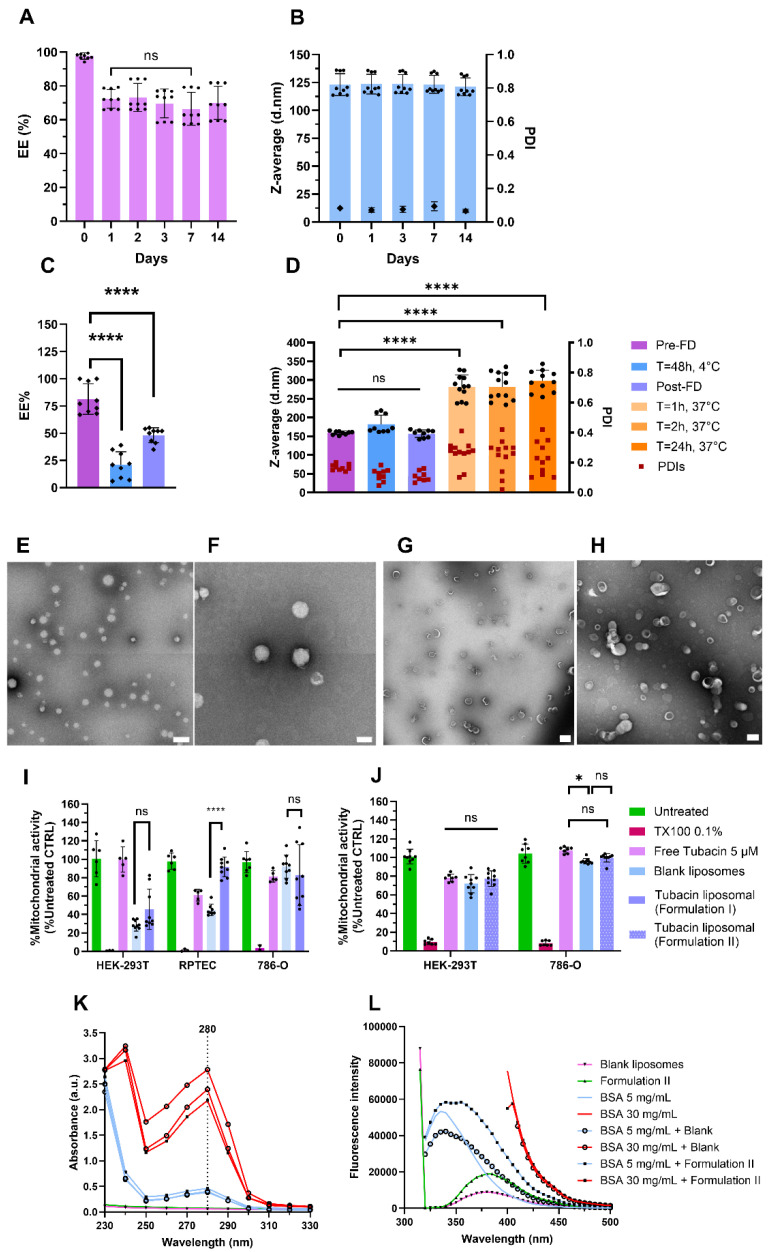
Formulations I and II characterization. (**A**) Encapsulation efficiency stability at 4 °C for 14 days for Formulation I. (**B)** Z-average and PDI stability of Formulation I at 4 °C obtained with DLS in batch mode. (**C**) Encapsulation efficiency of Formulation II pre-freeze-drying (FD), during storage for 48 h at 4 °C, and post-FD. (**D**) Z-average and PDI stability of Formulation II pre-FD, after 48 h of storage at 4 °C, post-FD, and in PBS at 37 °C for 1, 2, and 24 h. (**E**,**F**) TEM images of Formulation I. Scale bars = 500 nm (**E**) and 200 nm (**F**). (**G**,**H**) TEM micrographs of Formulation II pre-FD (**G**) and post-FD (**H**). Scale bars = 500 nm (**G**) and 200 nm (**H**). Mitochondrial activity of Formulation I (**I**) and Formulation II (**J**) on different renal cell lines after 24 h of treatment. An albumin interaction study with Formulation II after one hour of incubation. (**K**) The absorbance spectrum of Formulation II and (**L**) fluorescence spectrum after excitation at 280 nm Data were normalized to untreated cell results. Data are represented as mean ± SD (N = 3). **** indicates *p* < 0.0001, * *p* < 0.005, and ns stands for non-significance.

For Formulation I, the initial EE% was 98% (N = 3) on the day of liposome preparation. However, by day 1 (24 h later), the EE% decreased to 70% but remained stable during storage at 4 °C up to 14 days (Figure 2A). The EE% stability was followed at 37 °C for 24 h and showed no release of tubacin (see Supplementary Information S2 and Supplementary Figure S3). Formulation I’s particles were 123 ± 10 nm in diameter with a PDI below 0.1 (N = 3); see Figure 2B. Consistent with DLS analysis, the AF4-MALS-DLS analysis revealed no significant changes in peak profiles during 15 days of storage at 4 °C; see Supplementary Figure S4. For Formulation II, the pre-FD encapsulation efficiency varied between 75 and 100% but, after 48 h of storage at 4 °C, the encapsulation dropped inconsistently between 10 and 40%. Post-FD formulations exhibited a consistent encapsulation efficiency of 50%; see Figure 2C. The Z-average and PDI were 159 ± 5 nm and 0.2, respectively (N = 3), as measured immediately after liposome synthesis, and remained stable both after 48 h of storage at 4 °C and following the freeze-drying process. However, upon incubation in PBS at 37 °C for 1 h, the Z-average increased twofold to 293 ± 31 nm, while PDI rose to 0.3. These values remained constant for at least 24 h of incubation at 37 °C (N = 3); see Figure 2D. Size and morphology were analyzed using TEM with negative staining. Particles were stained with uranyl acetate. Both formulations presented spherical particles with low polydispersity (Figure 2E). The liposomes of Formulation I were unilamellar and the bilayer was visible (Figure 2F). TEM images revealed spherical and monodisperse liposomes of Formulation II, with no noticeable changes in morphology during the freeze-drying process (Figure 2G,H).

For Formulation I, WST-1 and LDH assays were conducted to assess cell metabolic activity as an indirect measure of cell viability. Toxicity was evaluated using both nonmalignant (HEK-293T and RPTEC) and cancerous cells 786-O (N = 3). As shown in Figure 2I, incubation of noncancerous cells with blank liposomes resulted in a decrease in mitochondrial activity. Mitochondrial activity in HEK-293T and RPTEC cells was measured at 28% and 45%, respectively. In contrast, 786-O cells were less affected, with 92% of mitochondrial activity remaining intact. Regarding cytotoxicity, as assessed by the LDH assay, incubation with blank liposomes induced 40% and 70% cytotoxicity in HEK-293T and RPTEC cells, respectively (Supplementary Figure S5). When tubacin was encapsulated into liposomes, mitochondrial activity was preserved at over 92%, with only 6% cytotoxicity observed in RPTEC cells. In contrast, HEK-293T cells exhibited greater sensitivity to the loaded liposomes, showing 40% mitochondrial activity and 50% cytotoxicity. Free tubacin did not demonstrate toxicity when incubated with HEK-293T cells. However, for RPTEC cells, the mitochondrial activity was reduced to 60% (Figure 2I), accompanied by 30% cytotoxicity (Supplementary Figure S5). The toxicity of blank liposomes toward noncancerous cells was unexpected, leading to the development of Formulation II to mitigate these toxicity levels.

For Formulation II, the biological activity of HEK-293T and 786-O cells was assessed again. Incubation with free tubacin, blank liposomes, and Formulation II resulted in 80% mitochondrial activity in HEK-293T cells. For 786-O cells, mitochondrial activity ranged between 90 and 100% under the same conditions (Figure 2J). Finally, Formulation II was incubated at 37 °C for 1 h to assess its interaction with albumin. The absorbance peak of albumin was detected at 280 nm and showed no shift when incubated with liposomes, regardless of whether they were loaded with tubacin or not (Figure 2K), indicating no binding between albumin and the liposomes. Similarly, the absence of fluorescence quenching further confirmed the results obtained from the absorbance spectrum (Figure 2L).

### 3.4. Formulation II Development

Formulation II was designed with reduced TPGS content and the incorporation of Kolliphor^®^ HS-15. Total lipid content was reduced by 4.3-fold, while drug-to-lipid molar ratio was increased by 3.6-fold. The lipid composition of Formulations I and II are described in Table 4.

The encapsulation of tubacin in Formulation II was not stable during storage at 4 °C in suspension. Consequently, liposomes were characterized before freeze-drying (pre-FD) and after (post-FD).

### 3.5. Tubacin Potency

**Figure 3:**
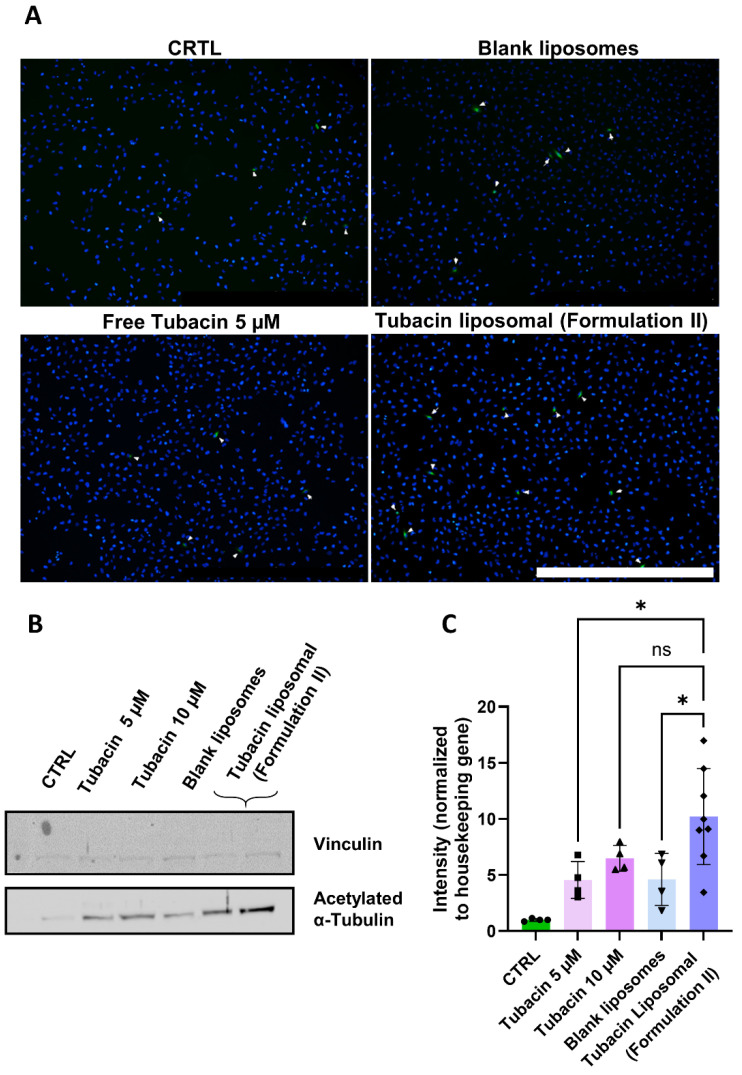
Effect of Formulation II on α-tubulin acetylation levels of 786-O cells. The treatment duration was 2 h. All experiments were performed under the same conditions. (**A**) Immunofluorescence stained for α-tubulin (green) and DAPI (blue). Scale bar = 1 mm. Magnification 4×. (**B**) Western blot was quantified in (**C**). Magnification 10×. Results are presented as mean ± SD (N = 3). The significance of * *p* < 0.005 and ns was determined with a one-way ANOVA test with post hoc Tukey’s multiple comparisons. The entire WB gel images can be found in Supplementary Figure S6.

Finally, to validate the potency of tubacin following liposome encapsulation and freeze-drying and, to assess its impact on α-tubulin acetylation, immunofluorescence staining (α-tubulin acetylation, green) was conducted. Tubacin-loaded liposomes were incubated with 786-O cells at a concentration of 5 µM. Higher levels of acetylation were observed with tubacin-loaded liposomes compared to its free form (Figure 3A).

Western blot analysis further supported these findings, revealing that the treatment with liposomal tubacin resulted in a staining intensity comparable to that of free tubacin at 10 µM (Figure 3B,C). Notably, liposomal tubacin at 5 µM exhibited superior efficacy compared to free tubacin at the same concentration.

## 4. Discussion

In this study, we developed, optimized, and characterized a liposomal formulation to enhance the drug delivery of tubacin. Critical parameters, including particle size, polydispersity index (PDI), encapsulation efficiency (EE%), and stability, were systematically evaluated. The usage of a Plackett–Burmann Design enables the rapid identification of key process parameters influencing the formulation.

TPGS demonstrated a positive effect on tubacin encapsulation, likely due to its surfactant properties, which enhance tubacin solubility in ethanol. Initially, DMSO was hypothesized to serve the same purpose, potentially increasing EE%. However, the observed negative correlation between DMSO and EE% suggests the opposite. The adverse effect of DMSO on tubacin encapsulation may be attributed to its permeability-enhancing properties. As an aprotic solvent, DMSO can induce water pores in DPPC bilayers, increasing membrane permeability and potentially leading to drug leakage [31]. Additionally, the negative effect of DPPC content on EE% may be linked to reduced membrane space for tubacin. Given the log P value of tubacin exceeding 7, it is likely integrated into the liposome’s phospholipid bilayer. The literature suggests that competition for bilayer space may arise between cholesterol and hydrophobic drugs [24]. However, in this study, we observed a positive correlation between cholesterol and encapsulation efficiency. A potential explanation could be that cholesterol enhances the packing density of liposomes, thereby reducing leakage of lipophilic drugs and improving drug encapsulation [32]. Other parameters were found to have no significant impact on EE% and were not further investigated.

Regarding particle size, this formulation was designed for intravenous administration. Size is a critical parameter for leveraging passive targeting through the enhanced permeation and retention (EPR) effect to reach solid tumors [33]. Consequently, liposome size should exceed the molecular weight cut-off for renal clearance (approx. 5 to 10 nm) to prolong circulation in the bloodstream [34]. However, there is no consensus in the literature on the upper size limit for optimal drug targeting. For example, Franco et al. suggested that a size between 100 and 150 nm is ideal for accessing solid tumor architecture [35,36], while other studies suggest an optimal size range between 100 and 200 nm [37]. In our study, tubacin stirring (or rotation) speed was found to increase particle size, whereas DMSO reduced it. Tubacin location within the lipid bilayer may contribute to an increased particle size. As previously discussed, DMSO’s disruption of lipid membranes could have led to tubacin leakage, resulting in the formation of smaller liposomes [38]. Stirring (or rotation speed) usually reduces particle size [39]. In our study, however, it led to an increased size. This contradictory result can be explained by the composition of the Plackett–Burman Design (PBD). Only three out of the nine formulations tested within the frame of the PBD are set at a rotation rate of 800 rpm. The limited number of formulations at this rotation speed creates a disparity that hinders a definitive conclusion.

The polydispersity index (PDI) is a critical parameter for assessing the stability of nanomedicines. A narrow size distribution is indicated by a PDI value between 0.1 and 0.25, while values above 0.5 suggest a broad size distribution. In this study, all PDI values fell within the narrow distribution range (below 0.5). This monodispersity could be likely attributed to the ethanol injection method (EIM) used for liposome synthesis. The injection speed was carefully controlled using an electronic pipette and remained consistent across all formulations prepared. Unlike the thin-film method, which often requires sonication to obtain a uniform size distribution, the EIM produces liposomes with low PDIs in a single step [36].

With the exception of Formulation 1, the sizes of all tested formulations were within the range of 100 and 160 nm. Consequently, encapsulation efficiency became the decisive parameter for the choice of optimal formulation. Formulation 4 met the target product profile with the highest EE% and particle size of 135 ± 8 nm. Further optimization was conducted by testing TPGS inputs of 3, 4, and 5 mg. The 5 mg input demonstrated excellent size stability at both 4 °C and 37 °C and was ultimately selected. Both DPPC and DMSO were found to reduce EE%; therefore, DPPC was maintained at the lowest tested input and DMSO was removed from the formulation. All other parameters remained consistent with those used for Formulation 4.

Next, temperature stability was assessed. In clinical practice, this formulation is intended to be stored at 4 °C and subsequently administered via systemic injection at 37 °C, necessitating size stability at both temperatures. AF4-DLS was chosen as the method to assess particle stability at 37 °C (body temperature) or 4 °C (fridge storage temperature). The formulation with the highest TPGS input (5 mg) showed the highest stability at both temperatures. The 5 mg TPGS was selected as the limit tested, as it corresponds to TPGS’s critical micellar concentration of 0.02% [40]. The highest TPGS content was advantageous for stability, likely due to the ability of non-ionic surfactants, such as TPGS, to form strong hydrogen bonds with water molecules. This hydration of the surfactant’s polar head group enhances its resistance to temperature variations [41].

Regarding storage stability, an initial decrease in EE% from 98% to 70% was observed after 24 h at 4 °C. This loss can likely be attributed to tubacin content that was loosely associated with the bilayer and, therefore, not fully encapsulated. This hypothesis was further supported by the release kinetics analysis, which demonstrated that the encapsulated tubacin content remained stable at 37 °C, indicating no significant drug release. The remaining 70% of encapsulated tubacin corresponds to a concentration of 43 µM, approximately 8-fold the in vitro concentration required for the C2 combination [9].

TEM imaging revealed a small number of particles appearing larger than others, which could affect size dispersity and contribute to an increase in the PDI. However, it is important to note that TEM can lead to artifacts, such as distortion during the drying process [42], which may influence the particle size.

In the next step, the toxicity of Formulation I towards both nonmalignant cell lines (HEK-293T and RPTEC) and cancerous cells (786-O) was evaluated. HEK-293T, a commonly used cell line created by the viral transformation of human embryonic kidney cells, serves as a healthy control in kidney-related research [43]. RPTEC, originating from renal proximal tubule epithelial cells, is also employed as a healthy control. The 786-O cell line, one of the first primary cell lines established for RCC studies, is extensively used in research focused on RCC [44]. Both the WST-1 and LDH assays indicated the toxicity of blank liposomes on nonmalignant cells. These results suggest that the reduced mitochondrial activity observed in the WST-1 assay is linked to a form of cell death, with RPTEC cells being more affected than HEK-293T cells. However, when tubacin is loaded in liposomes, toxicity in RPTEC cells diminishes compared to HEK-293T. Unexpected toxicity is observed in nonmalignant cells following exposure to Formulation I. Given that DPPC and cholesterol are endogenous to the body [45]. TPGS, a vitamin E derivative conjugated with a PEG moiety, is widely employed as a permeation enhancer in transdermal drug delivery and is recognized by the FDA as a safe pharmaceutical excipient. Its amphiphilic properties facilitate the solubilization of hydrophobic compounds, increasing encapsulation efficiency, as confirmed by the PBD-based results. The PEG moiety increases the particle’s hydrophilicity, thus leading to prolonged particle circulation by reducing immune clearance. Particles formed with TPGS had superior cellular uptake across several cancer cell lines (e.g., C6 glioma and MCF-7) [46]. This can be explained by the presence of vitamin E receptor-mediated internalization enhancing anticancer effect [40,47]. As a non-ionic surfactant, TPGS is considered less toxic than ionic surfactants whose toxicity has been reported [47]. To our knowledge, this is the first report of TPGS-induced toxicity. To address this, we developed Formulation II to reduce toxicity levels.

Formulation II was prepared following insights gained from the PBD and the literature on systemically administered liposomal formulation for anticancer purposes. To enhance stability and minimize drug leakage, DPPC was replaced by DSPC, which presents a higher phase transition temperature [48]. Cholesterol content was fixed at 30 %mol, which is a threshold frequently reported to be optimal to secure bilayer stability [24]. Finally, overall lipid concentration was lowered from 2.88 to 0.71 mg/mL to improve the drug-to-lipid ratio and potentially increase tubacin encapsulation yield. As a functional excipient, TPGS was conserved to develop a new tubacin liposomal formulation. The complete removal of TPGS led to unstable particles and precipitation. Given its toxicity towards healthy renal cells, TPGS levels were reduced and Kolliphor^®^ HS-15 was added. This FDA-approved surfactant used for both ophthalmic and parenteral formulations has a CMC value between 0.005% and 0.02% [49]. It consists of a mixture of mono- and di-ester polyglycols of 12-hydroxystearic acid with a fraction of 30% free polyethylene glycol. Compared to other non-ionic surfactants such as Tweens, intravenous administration of Kolliphor^®^ HS-15 leads to lower hemolysis and irritancy [50]. Solubility assays of tubacin in Kolliphor^®^ solutions were conducted to identify optimal tubacin solubilization leading to Kolliphor^®^ HS-15 concentration fixed at 0.04 mg/mL.

Formulation II exhibited drug leakage during fridge storage at 4 °C and an increase in particle sizes from 150 nm to 250 nm in PBS at 37 °C. These instabilities could be explained by two mechanisms. Firstly, cholesterol content within the bilayer is reduced by 50% compared to Formulation I. Cholesterol is well known for its role as a stabilizer, reducing the fluidity of the bilayer and minimizing drug leakage [51]. Secondly, TPGS concentration was fivefold, which likely impacts particle stability as previously discussed. To address these stability issues, Formulation II was therefore freeze-dried.

However, the freeze-drying process led to 50% of drug loss, though the final drug content remained consistent across batches post-FD. Final drug encapsulation equals 34 µM, which represents an approximately 7-fold higher concentration required in the C2 drug combination [9]. With a total lipid concentration 4-fold lower, Formulation II presented more efficient drug loading, exhibiting a 3.5-fold increase in the drug-to-lipid ratio even post-FD. This improved yield indicates a more efficient encapsulation process. Z-average and PDI of Formulation II were consistent before and after freeze-drying and were maintained during storage in a liquid state for 48 h at 4 °C. However, at 37 °C in PBS, particle sizes doubled, suggesting a temperature-dependent instability. This effect could be likely explained by the reduction in TPGS content, rendering liposomes more sensitive to temperature.

To assess potential toxicity, we performed evaluations in both nonmalignant and cancerous cells with no detectable cytotoxic effects observed. The absence of anticancer activity in malignant cells is probably due to the use of tubacin at low-dose monotherapy.

Indeed, in a previous study, the viability of 786-O cells exposed to 5 µM tubacin was reduced by 25%. However, when combined with tacedinaline, dasatinib, and erlotinib, cell viability dropped by 95%, suggesting a lack of anticancer activity of tubacin as a single agent at the tested dose.

Additionally, no interaction with albumin was observed. This result is important as nanoparticles entering the bloodstream are rapidly coated with plasma proteins. The effect forms a “biomolecular corona”, altering the particle surface and thus the “biological identity” of the particles. This altering can significantly influence the nanoparticle fate, potentially increasing or decreasing blood circulation time [52]. The nanoparticle features, such as charge and surface functional groups, dictate the protein composition of this corona. In this study, albumin interaction was specifically investigated due to its abundance of plasma and its role in drug transport [53,54]. The lack of interaction observed may be explained by the liposomes being PEGylated. Indeed, the primordial role of albumin is to bind to hydrophobic drugs to increase their hydrophilicity. However, as a hydrophilic moiety, PEGylation creates a steric barrier, theoretically reducing protein adsorption. It is disputed whether PEGs presence increases blood circulation, but several studies answered positively to that question [55]. This effect is hypothesized to result from the inhibition of proteins involved in renal clearance by PEGs [56]. The absence of albumin interaction does not necessarily guarantee nanoparticle stability. In certain cases, albumin binding to nanoparticles can enhance tumor accumulation. Some cancers, e.g., breast, melanoma, head, and neck, overexpress SPARC receptors that facilitate the cellular uptake of particle–albumin complexes [54].

Finally, the potency of tubacin was evaluated with immunofluorescence. Tubacin promotes the acetylation of the Lys40 site on α-tubulin, thereby stabilizing microtubules [14]. The experimental conditions included a control (CTRL), consisting of liposome external buffer and RPMI medium, and a representative amount of DMSO present in the free tubacin at concentrations of 5 and 10 µM used as positive control. Blank liposomes were included to ensure that any observed activity was drug-specific. Both immunofluorescence and Western blot analysis showed higher acetylation levels with tubacin-loaded liposomes compared to its free form. These findings may be attributed to several factors. Free tubacin is highly hydrophobic and may precipitate in cell media, whereas tubacin encapsulated in liposomes remains soluble and, hence active. Secondly, differences in release kinetics may play a role. The potency of tubacin was assessed after a 2 h treatment period, during which free tubacin was immediately available for cellular uptake. In contrast, liposomal tubacin may experience a more gradual release and uptake by cells. We propose a dual-phase release mechanism to explain these observations: an initial rapid release of tubacin is followed by a sustained release of the remaining encapsulated fraction. This biphasic release profile likely accounts for the enhanced α-tubulin acetylation observed at 2 h compared to free tubacin at equivalent concentrations (5 µM), as the prolonged exposure maintains effective intracellular drug levels. The delayed release from liposomes may provide more sustained target engagement than the bolus delivery of free drug, despite identical nominal concentrations.

## 5. Conclusions

The initial formulation demonstrated excellent stability with no detectable drug release at 37 °C; however, it showed poor tolerability in healthy renal cell lines. To address this limitation, we developed Formulation II by integrating findings from our Plackett–Burman Design with established liposomal formulation strategies from the literature. This optimized formulation incorporated modifications in the amount of the main lipid component reduced cholesterol and TPGS content, and an addition of non-ionic surfactant, Kolliphor^®^ HS-15. This revised formulation required freeze-drying for storage, which resulted in drug loss during the process. However, despite a lower tubacin content load, Formulation II had a better drug-to-lipid ratio and was better tolerated by healthy cell lines. Finally, tubacin potency after freeze-drying was conserved.

## Figures and Tables

**Table 1:** Plackett–Burmann Design table and results.

Formulation n°	Temp. (°C)	Rotation Speed (rpm)	Rotation Time (min)	Tubacin Input (µg/mL)	DMSO (µL)	DPPC (%)	Cholesterol (%)	TPGS (%)	EE (%)	Size (nm)	PDI
1	50	800	5	50	200	49.6	37.7	12.7	18 ± 8	236 ± 24	0.174
2	60	600	10	20	200	21.8	68.9	9.30	27 ± 2	135 ± 4	0.09
3	50	600	10	20	500	39.6	30.1	30.3	15 ± 6	120 ± 19	0.165
4	50	600	5	50	500	18.4	58.2	23.5	38 ± 13	136 ± 8	0.067
5	60	600	10	50	200	39.6	30.1	30.3	37 ± 18	163 ± 8	0.066
6	50	600	5	20	200	37.2	47.0	15.8	30 ± 4	148 ± 3	0.097
7	60	800	5	20	200	27.3	51.8	20.9	38 ± 5	149 ± 5	0.020
8	60	800	5	20	500	28.2	35.7	36.1	28 ± 5	130 ± 4	0.092
9	60	600	5	50	500	31.7	60.2	8.10	9.0 ± 3	141 ± 9	0.091

DPPC, cholesterol, and TPGS are displayed as molar ratio percentages. Results are shown ± SD, N = 3.

**Table 2:** Summary of every variable, their levels of significance (*p*-value) on EE%, particle size, and PDI, and type of impact on responses (positive/negative).

	EE%	Size	PDI
Independent variables	***p*** **-value**		
**Temperature**	n.a.	0.079	0.052
**Rotation speed**	n.a.	**0.026/Positive**	0.067
**Tubacin concentration**	0.106	**0.016/Positive**	n.a.
**DPPC**	**0.029/Negative**	n.a.	n.a.
**Cholesterol**	0.119	n.a.	n.a.
**TPGS**	**0.007/Positive**	n.a.	0.153
**Loading time**	n.a.	n.a.	0.103
**DMSO**	**0.009/Negative**	**0.027/Negative**	0.191

**Table 3:** Formulation I and its selected parameters.

Formulation	Temperature	Rotation (rpm)	Rotation Time (min)	Tubacin Input (mg/mL)	DPPC Input (mg)	Cholesterol Input (mg)	TPGS Input (mg)
I	50	600	5	0.05	3	5	3

**Table 4:** Composition of Formulations I and II.

	Compound	Concentration (mg/mL)	Molar Ratio	%mol in the Bilayer	Total Lipids (mg/µmol)	Final Drug Content (µM/µmol)	Drug-to-Lipid Molar Ratio
Formulation I	DPPC	0.6	1.0	20	13/20.32	48.3/0.243	0.012
	Cholesterol	1.0	3.1	64			
	TPGS	1.0	1.6	16			
Formulation II	DSPC	0.3	1.0	48	3/4.06	34.5/0.173	0.043
	Cholesterol	0.1	0.1	31			
	TPGS	0.2	0.63	16			
	Kolliphor^®^ HS-15	0.04	0.32	5			

## Data Availability

The data supporting this work are accessible at: Schelker, Cindy; Revaclier, Léa; Borchard, Gerrit; Nowak-Sliwinska, Patrycja (2025), “Tubacin encapsulation”, Mendeley Data, V2, doi: 10.17632/zgcgvkv42d.2.

## Supplementary Materials

The following supporting information can be downloaded at: https://www.mdpi.com/article/10.3390/pharmaceutics17040491/s1, Information S1: Cytotoxicity (LDH); Information S2: In vitro release studies; Table S1: Example of table with data extracted from chromatogram shown in Supplementary Figure S1; Table S2: Freeze-drying protocol; Table S3: Independent variables and levels screened using the Plackett-Burman Design; Table S4: Parameters used during the elution step; Figure S1: Example of a chromatogram of tubacin separation with UPLC-UV technique. The peak at 5.610 corresponds to tubacin. The peak at 6.400 corresponds to Triton X-100; Figure S2: Example of a calibration curve for tubacin quantification in DMSO:NaCl 4:1 *v*/*v*. R2 = 0.9983 and the linear equation used for calculations was y = 94,210,087x − 15,730. n = 3; Figure S3: Encapsulation efficiency stability of Formulation I in PBS at 37 °C. N = 3; Figure S4: AF4-MALS-DLS fractogram of Formulation I at day 0 (day of synthesis) and day 15, storage at 4 °C. N = 3; Figure S5: Cytotoxicity of Formulation I. Data were normalized to untreated cell results or those treated with 0.1% Triton X-100 in medium for LDH assays, respectively. Data are represented as mean ± SD (N = 3). **** indicates *p* < 0.0001, and ns indicates non-significance; Figure S6: Original western blots of the gene of interest acetylated α-Tubulin (top membrane) and housekeeper gene vinculin (bottom membrane); Figure S7. The Pareto chart represents the different parameters and their effect on the EE% from the highest to the lowest contribution. Factors depicted in orange have a positive effect (increase in EE%) whereas factors depicted in blue have a negative effect (decrease in EE%); Figure S8. The Pareto chart representing the effect of each factor on particle size, from highest to lowest contribution. Factors depicted in orange have a positive effect (increase in particle size) whereas factors depicted in blue have a negative effect (decrease in particle size). Tubacin concentration, rate of rotation, and amount of DMSO bars are above the t-value limit, meaning they are considered significant variables (*p* < 0.05). Figure S9. Pareto charts represent each factor's effect on the PDI from highest to lowest contribution. Factors depicted in orange have a positive effect (increase in PDI) whereas factors shown in blue have a negative impact (decrease in PDI).
