# Supplementary material for: Liposomal Tubacin: Strategies for the Formulation of a Highly Hydrophobic Anticancer Drug
Source: Pharmaceutics. 2025 Apr 8;17(4):491. doi: 10.3390/pharmaceutics17040491 (PMC12030124; doi:10.3390/pharmaceutics17040491)
Supplement: Supplementary file 1 [file pharmaceutics-17-00491-s001.zip › pharmaceutics-3520570-supplementary.pdf]

pharmaceutics

Supplementary Material

Liposomal Tubacin: Strategies For The Formulation of A Highly Hydrophobic Anticancer Drug

Material and methods (supplementary)

Supplementary Information S1: Cytotoxicity (LDH)

HEK-293T and 786-O cells were seeded at an initial density of $5 \cdot 10^5$ cells per well in 96-well plates and allowed to attach for 24h. The cells were treated with 0.9% NaCl in medium (CTRL), blank liposomes, free tubacin, or liposomal tubacin (Formulation I) and incubated for 24 hours. The activity of the enzyme lactate dehydrogenase (LDH) was assessed using an LDH cytotoxicity assay kit following the manufacturer's protocol (Thermofisher Scientific, Waltham, MA). A 50 µL aliquot of medium was collected from each sample and transferred to a new 96-well plate. The cells were lysed using 10 µL of lysate buffer from the kit for 45 min at 37°C in a humidified atmosphere with 5% CO_2. UV absorbance was measured at λ = 490 nm using a microplate reader (BioTek Instruments, Sursee, Switzerland). Triton 0.1% X-100 was used as a positive reference corresponding to 0 % viability. Equation 1 was used to calculate the cytotoxicity of every sample:

$$\%Cytotoxicity = \frac{Absorbance\ (sample)}{Absorbance\ (Triton\ X100\ 0.1\%\ sample)} \times 100 \qquad (1)$$

Supplementary Information S2: *In vitro* release studies

Conventional release study methods typically involve separating free and encapsulated forms before quantification. Due to the insolubility of tubacin limits, conventional release study methods, such as dialysis are ineffective. Instead, we quantified the encapsulated tubacin after removing its free form by diluting 1 mL of liposome suspension in 9 mL of PBS and incubating it under shaking at 37°C. At specific time points, 500 µL aliquots were transferred to centrifugal filters (Amicon Ultra 0.5 centrifugal filter unit, MWCO: 100 kDa). Centrifugation was carried out at 8000 rpm for 50 min at 4°C. The liposome suspension was reconstituted to its initial volume with phosphate-buffered saline (PBS), 1 µL of Triton X-100 was added, and the mix was vortexed for 10 seconds. Complete liposome disruption was assured using an ultrasound bath at 50°C for 15 minutes. The final mixture was analyzed by UPLC.

Supplementary Table S1: Example of table with data extracted from chromatogram shown in **Supplementary Figure S1**.

	Retention Time	Area	% Area	Height
1	5.610	689910	31.01	156624
2	6.400	1534876	68.99	247656

Supplementary Table S2: Freeze-drying protocol

Step	Process	°C	Pressure (mbar)	Time (hh:mm)
1	Shelves preparation	-22	/	03:00
2	Freezing	-40	/	00:05
3	Condenser preparation	/	/	00:50
4	Chamber vacuum		0.3	02:00
5	Primary drying	-40	0.3	25:00
6	Primary drying	-20	0.3	05:00
7	Primary drying	-10	0.3	03:00
8	Secondary drying step	22	/	04:00
9	End of cycle	/	/	/

Supplementary Table S3: Independent variables and levels screened using the Plackett-Burman Design.

Factor coding	Variable	Units	Level (-1)	Level (+1)
A	Temperature	°C	50	60
B	Stirring speed	Rpm	600	800
C	Loading time	min	5	10
D	Tubacin concentration	µg/ml	20	50
E	DPPC	mg	3	5
F	Cholesterol	mg	2	5
G	TPGS	mg	1	3
H	DMSO	ml	0.2	0.5

Supplementary Table S4: Parameters used during the elution step.

Step	Time (min)	Cross-flow (ml/min)	Type	Exponent
1	30	1.00	Linear	1.0
2	5.0	0.19	Power	0.8
3	5.0	0.10	Power	0.8
4	8.0	0.06	Power	0.8
5	8	0.037	Power	0.8

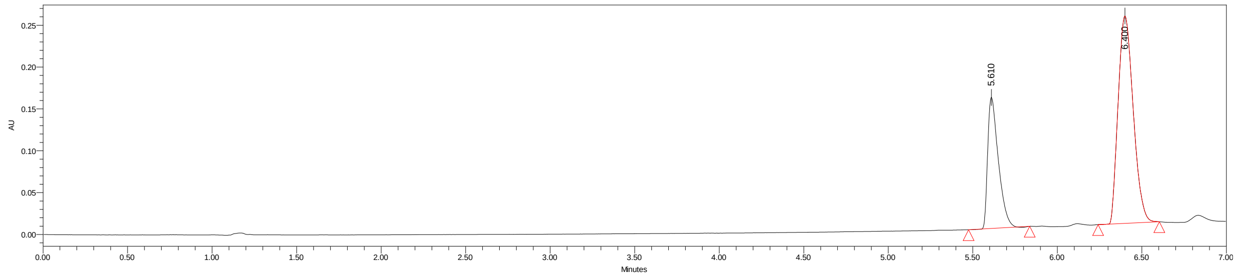

Supplementary Figure S1. Example of a chromatogram of tubacin separation with UPLC-UV technique. The peak at 5.610 corresponds to tubacin. The peak at 6.400 corresponds to Triton X-100.

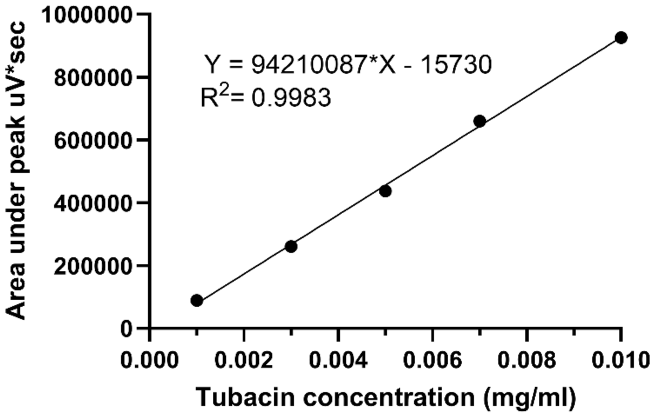

Supplementary Figure S2. Example of a calibration curve for tubacin quantification in DMSO:NaCl 4:1 v/v. R^2=0.9983 and the linear equation used for calculations was y=94210087x-15730. n=3.

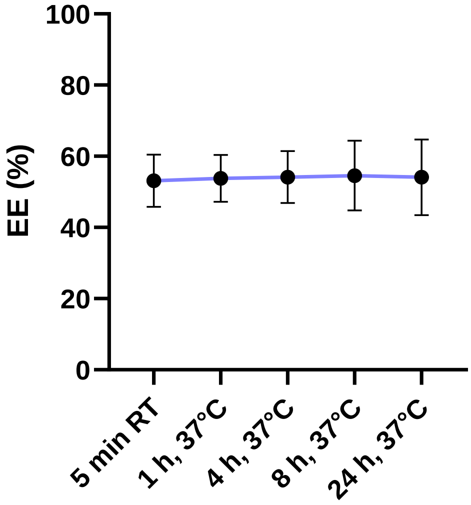

Supplementary Figure S3. Encapsulation efficiency stability of Formulation I in PBS at 37°C. N=3.

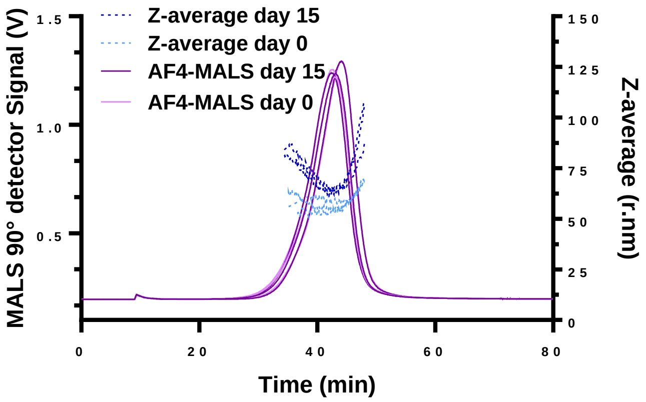

Supplementary Figure S4. AF4-MALS-DLS fractogram of Formulation I at day 0 (day of synthesis) and day 15, storage at 4°C. N=3.

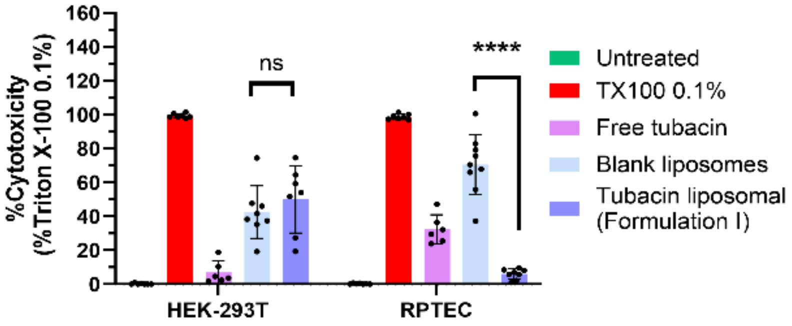

Supplementary Figure S5. Cytotoxicity of Formulation I. Data were normalized to untreated cell results or those treated with 0.1% Triton X-100 in medium for LDH assays, respectively. Data are represented as mean ± SD (N=3). **** indicates $p<0.0001$, and ns indicates non-significance.

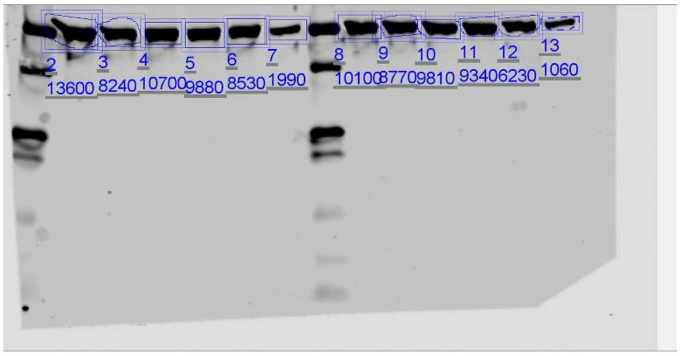
2 3 4 5 6 7 8 9 10 11 12 13
13600 8240 10700 9880 8530 1990 10100 8770 9810 9340 6230 1060

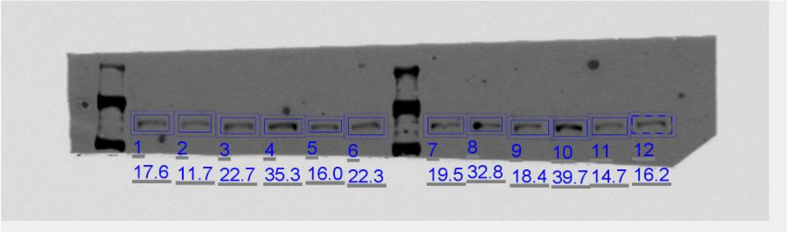
1 2 3 4 5 6 7 8 9 10 11 12
17.6 11.7 22.7 35.3 16.0 22.3 19.5 32.8 18.4 39.7 14.7 16.2

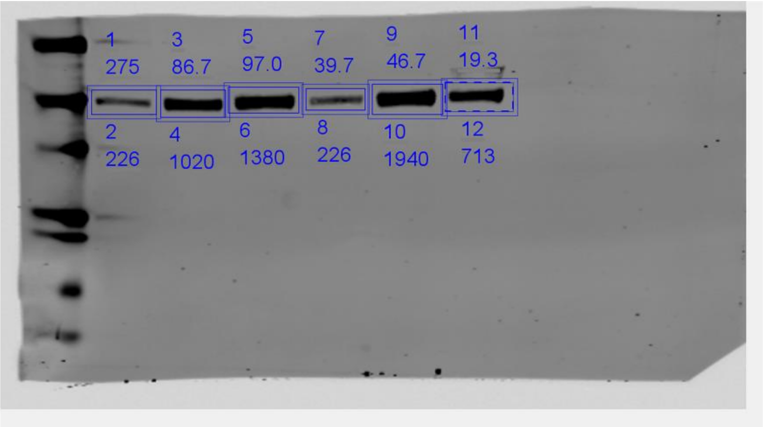
1 3 5 7 9 11
275 86.7 97.0 39.7 46.7 19.3
2 4 6 8 10 12
226 1020 1380 226 1940 713

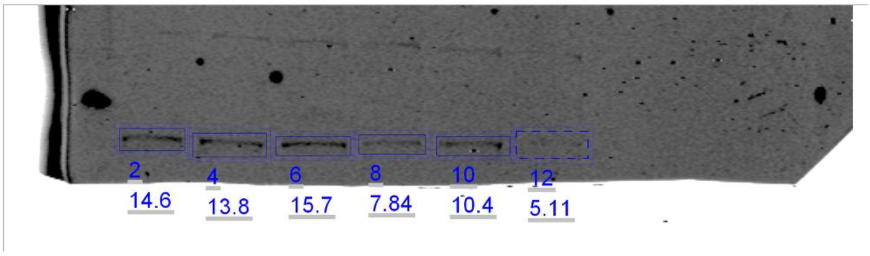
2 4 6 8 10 12
14.6 13.8 15.7 7.84 10.4 5.11

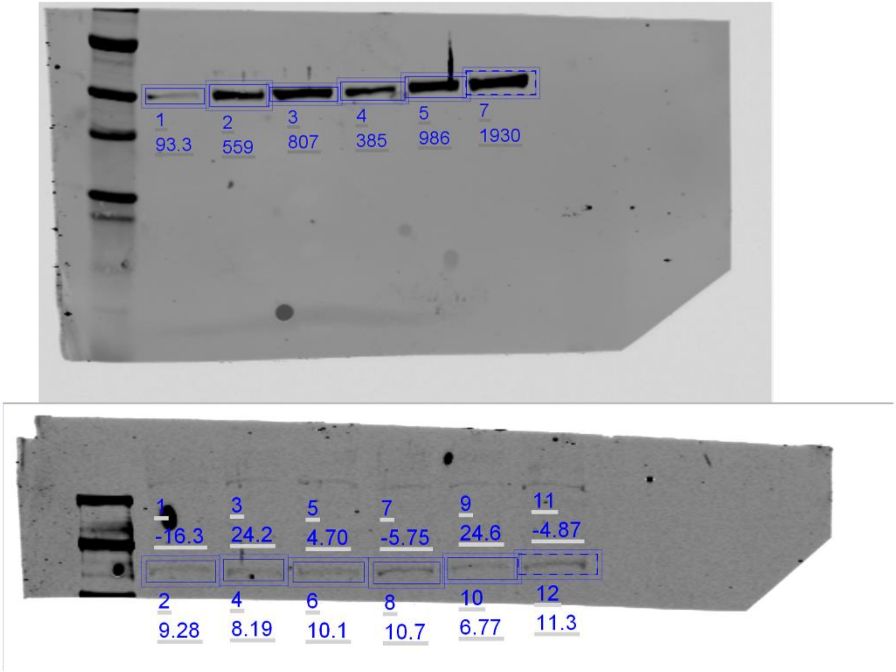

Supplementary Figure S6: Original western blots of the gene of interest acetylated α-Tubulin (top membrane) and housekeeper gene vinculin (bottom membrane).

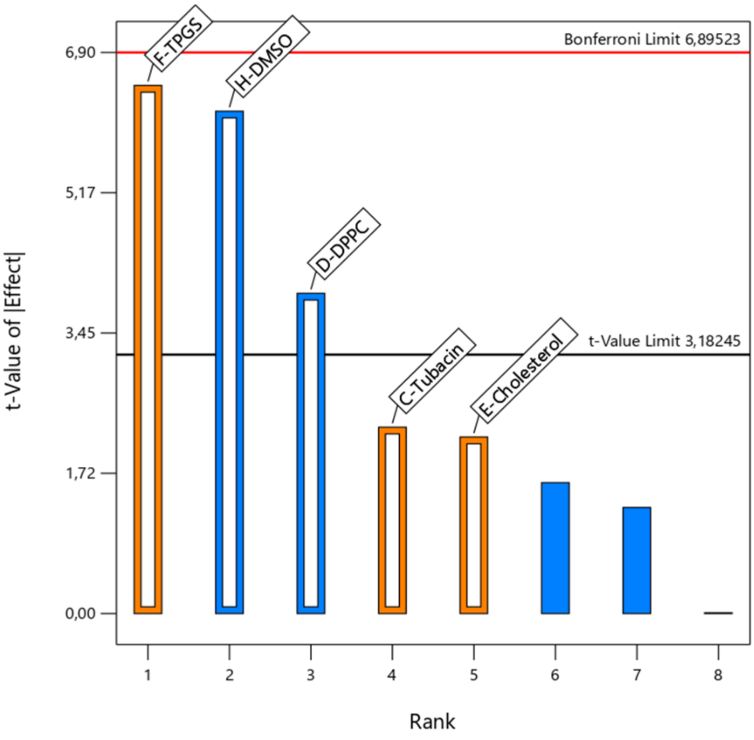

Supplementary Figure S7. The Pareto chart represents the different parameters and their effect on the EE% from the highest to the lowest contribution. Factors depicted in orange have a positive effect (increase in EE%) whereas factors depicted in blue have a negative effect (decrease in EE%).

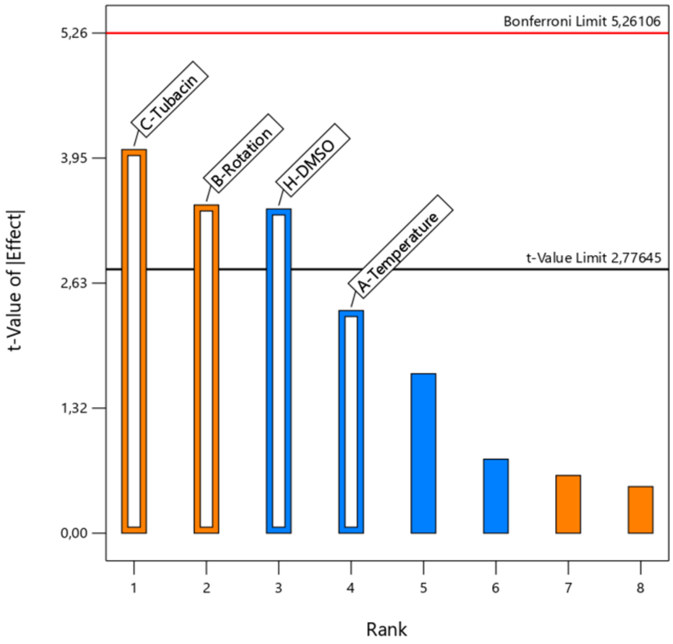

Supplementary Figure S8. The Pareto chart representing the effect of each factor on particle size, from highest to lowest contribution. Factors depicted in orange have a positive effect (increase in particle size) whereas factors depicted in blue have a negative effect (decrease in particle size). Tubacin concentration, rate of rotation, and amount of DMSO bars are above the t-value limit, meaning they are considered significant variables ($p<0.05$).

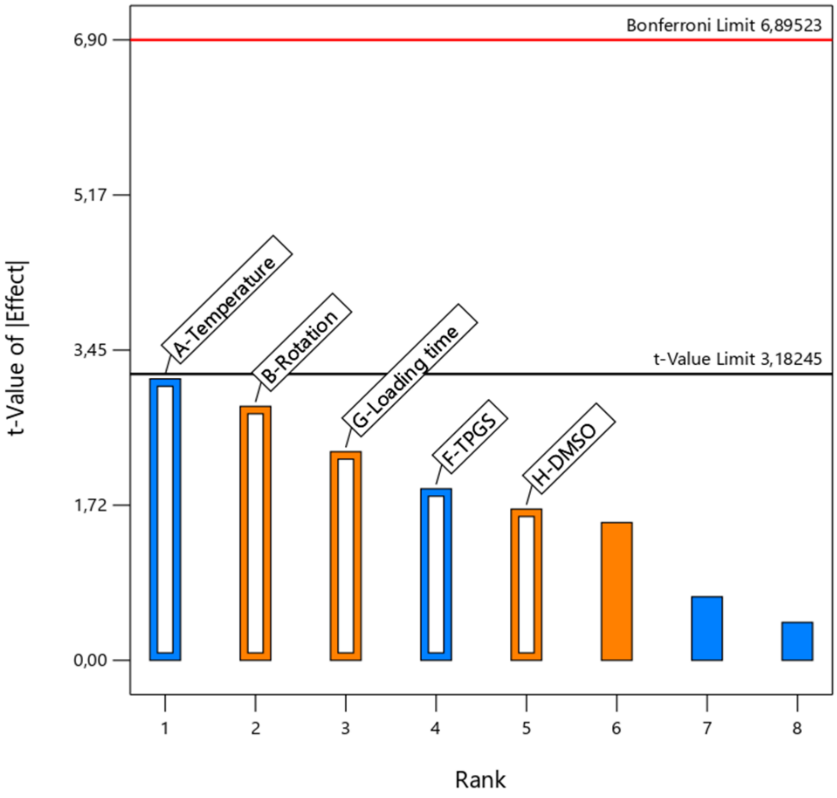

Supplementary Figure S9. Pareto charts represent each factor's effect on the PDI from highest to lowest contribution. Factors depicted in orange have a positive effect (increase in PDI) whereas factors shown in blue have a negative impact (decrease in PDI).

Disclaimer/Publisher's Note: The statements, opinions and data contained in all publications are solely those of the individual author(s) and contributor(s) and not of MDPI and/or the editor(s). MDPI and/or the editor(s) disclaim responsibility for any injury to people or property resulting from any ideas, methods, instructions or products referred to in the content.
